# Experiences and recommendations of veterinarians for the Dutch disciplinary system—a survey‐based study

**DOI:** 10.1002/vro2.67

**Published:** 2023-06-25

**Authors:** Iaira E. Boissevain, Anthonie W. Jongbloed, Franck L. B. Meijboom, Jan Willem Hesselink, Paul J. J. Mandigers

**Affiliations:** ^1^ Department of Clinical Sciences Faculty of Veterinary Medicine, Utrecht University Utrecht The Netherlands; ^2^ Department of Population Health Sciences, Division of Animals in Science and Society Faculty of Veterinary Medicine, Utrecht University Utrecht The Netherlands; ^3^ Utrecht University School of Law, Molengraaff Institute for Private Law Utrecht The Netherlands

## Abstract

**Introduction:**

In the Netherlands, the veterinary disciplinary system is governmentally executed and was originally designed to provide an educational effect on veterinarians as part of maintaining quality standards.

**Methods:**

Over 900 veterinarians were questioned (20% of the total number of veterinarians working in veterinary medicine in the Netherlands). It was ascertained whether or not they were aware of the disciplinary system, if it affected their way of working and what impact it had on their way of working after having faced a disciplinary case. Respondents were given the opportunity to express their opinions about the system and possible improvements.

**Results:**

The risk of complaints was found to be significantly greater when a veterinarian owned a practice compared to veterinarians who were employees. Veterinarians with their own practice were generally older (male) veterinarians. Whether this was a direct effect or just the effect of having a longer career could not be answered. Multiple disciplinary procedures appeared to have no influence. In 13%, veterinarians indicated that the disciplinary system had led to a more defensive way of practicing medicine to avoid complaints.

**Discussion and conclusions:**

Most veterinarians supported a disciplinary system as a tool for maintaining and improving the integrity and reputation of the profession as a whole. Recommendations to improve were (1) shortening the length of the procedure, (2) screening for validity, (3) using online systems for communication with the disciplinary council, (4) the option of mediation before getting involved in a full procedure and (5) instituting a complaint fee.

## INTRODUCTION

In most countries, governments have either instituted statutory bodies such as the Royal College of Veterinary Surgeons (RCVS) or incorporated a veterinary disciplinary system (DS) to promote the integrity and reputation of the profession and to offer an objective platform for complaints.^1^, ^2^, ^3^ In the Netherlands, the government has established a DS; it is not comparable with a statutory body such as the RCVS. All disputes are settled in accordance with a code of conduct drawn up by the Royal Dutch Veterinary Association Koninklijke Nederlandse Maatschappij voor Diergeneeskunde (KNMvD). The KNMvD has no influence on the way the government DS operates. Furthermore, the code of conduct only applies to members of the KNMvD and membership is voluntary. Veterinarians who feel that a colleague is not working at the highest professional standard can only complain through the KNMvD, provided that they are both members. If one of them is not a member, this ‘code of conduct’ cannot be used to file a complaint. The DS can only be used by owners and the so‐called civil servant (CS). The DS consists of two bodies: veterinary disciplinary council (VDC) and veterinary appeal council (VAC). All complaints are submitted to the VDC. If a ruling has been made and either one of the two parties disagrees, then the case can be submitted to the VAC.^1^ When the Dutch government created the DS, its primary function was only to maintain veterinary quality for animal welfare and food safety. However, it quickly became a platform for client complaints (CCs); this was not the original intention of the Dutch government.^1^, ^2^

Owners may not always realise the effect their complaints may have on the individual veterinarian. Several publications have reported on the huge impact of CCs on veterinarians and their colleagues.^4^, ^5^, ^6^ In human and veterinarian medicine, CCs contribute to psychological effects such as depression, burnout, anxiety and even suicidal ideation.^7^, ^8^ Furthermore, CCs may cause health problems such as cardiovascular disease, insomnia, headaches, anger and relationship problems.^4^, ^9^ Often these CCs and disciplinary procedures are so traumatic that healthcare workers become ‘second victims’.^10^ Studies in human medicine have reported that this may lead to a more defensive practice of medicine in which health workers try to avoid similar patients, become less communicative, are less focused on the patients’ problems and do more and often unnecessary diagnostic investigations.^9^, ^11^, ^12^, ^13^

The Dutch veterinary DS operates differently compared to other veterinary DSs, including those in the UK^3^ and USA,^6^ where complaints are first evaluated by a committee and only taken into consideration after careful scrutiny. The Dutch VDC and VAC take nearly all complaints into consideration.^1^ This places a huge burden on practicing veterinarians because they are confronted with any complaint whatsoever, automatically leading to a full disciplinary hearing. The hearings may have a major impact on their daily work and life.^5^, ^6^, ^7^, ^8^, ^14^ Whether there is a causal relation is unclear; one study reported that nearly 17% of recent (or new) graduates left private practice within 5 years of graduation.^15^ Another study reported that around 23% of older veterinarians opted for early retirement.^16^

Given previous studies,^4^, ^5^, ^6^, ^7^, ^8^, ^9^, ^12^ we formulated a number of questions concerning the Dutch DS that have been in place since 1993. For this, a web‐based survey study was designed. The aims of this study were to investigate veterinarians’ experiences with the DS, how it affected their way of working prior to having faced a disciplinary case (DC) or after facing a DC, which veterinarians were confronted the most with DCs, what they think of the process and what recommendations they have to improve it.

## MATERIALS AND METHODS

### Design

A web‐based questionnaire was designed using Qualtrics Experience Management (www.qualtrics.com/). The questionnaire consisted of 36 multiple choice questions and several open‐ended questions devised by two of the authors. The questions were designed using two studies investigating the impact of disciplinary procedures on human healthcare workers in the UK (which addressed the impact of complaint procedures on welfare, healthcare and clinical practices)^9^ and the Netherlands (which was a qualitative interview study investigating the impact of disciplinary processes).^17^

The questionnaire was separated into four sections (see Supporting Information S1 translated from Dutch into English). The first addressed demographic data, including gender, graduation year, in which field of veterinary medicine they were active and employment status. The second focused on whether they were aware of the existence of the VDC and VAC; also did this knowledge have any influence on their way of practicing. The third part was only open to veterinarians against whom a DC had been filed. The questions focused on what their experience was, what the verdict was, whether they agreed with the verdict and if they felt they had learned anything from it. The fourth part was open to all participants and comprised so‐called statement questions. All participants consented to participate in the study at the beginning of the questionnaire. They were informed that we would not store their IP addresses or any other information that could be used to identify them as a person. They were asked to fill in the questionnaire only once. All responses would be anonymous and untraceable; they were marked with a unique code.

### Participants

Veterinarians working in any type of field of veterinary medicine in the Netherlands were invited to participate in a web‐based questionnaire. Invitations were sent out using (1) communications of the KNMvD and (2) direct e‐mailing using the directory of the University Clinic of the Faculty of Veterinary Medicine, Utrecht University. Additionally, it was published on Facebook and LinkedIn. Invitations were sent to all veterinarians registered in the Netherlands. The majority of the possible participants were Utrecht graduates (around 80%) or Ghent graduates (around 20%).^16^, ^18^ The questionnaire was sent out twice to collect as many responses as possible. The first announcement was October 2017 for a period of 1.5 months. The second announcement was October 2022 for the same time period. In October 2022, the total number of registered veterinarians in the Netherlands was approximately 6000, with approximately 4500 practicing veterinarians.^18^ All others were employed by pharmacy, government, etc.^18^ The goal was to collect at least 450 responses.

### Analysis

The registered veterinarians all graduated between 1950 and 2022. This timeline was divided into three periods: (1) before 1990, as this was the year the DS was announced in the Netherlands; (2) 1991–2005, in which there was limited attention to the DS within the Dutch veterinary curriculum; and (3) 2006 onwards, in which the subject was thoroughly embedded into the Dutch curriculum.

The group of respondents was divided by age. Five age groups were created: group 1, less than 30 years of age; group 2, 30–39 years of age; group 3, 40–49 years of age; group 4, 50–59 years of age; and group 5, more than 60 years of age. The results were analysed with version 29 of the IBM SPSS software (Cary, NC, USA) and included descriptive statistics and chi‐squared tests. The results were considered statistically significant if the *p*‐value was less than 0.05.

## RESULTS

In total, 905 veterinarians (313 male [35%], 586 female [65%] and six unknown) completed the questionnaire, with 178 (21%) graduating before 1991, 342 (38%) between 1991 and 2005, and 367 (41%) after 2005. Nine veterinarians did not answer the date of graduation question. In the first period, the graduates were predominantly male and in the last period, they were predominantly female (*χ* ^2^[df = 2, 161], *p* < 0.001).

There were 28 veterinarians no longer working (17 retired, three incapacitated to work and eight had switched to another profession). Hence, 877 (97%) were still working within the field of veterinary medicine. In total, 688 out of 905 (76%) were working as companion animal practitioners at the time of this study, 27 (2.9%) worked with large animal livestock, 41 (4.5%) worked with horses and 91 (10%) worked with a mixed practice. The remaining 58 (6.4%) worked within different branches of veterinary medicine, such as the food industry, pharmacy, government and education. There were 66 with two jobs at the same time. Of the 905 respondents, 170 (19%) had worked in a different branch of veterinary medicine. The majority of these 170 individuals (114 persons [13%]) changed their work focus to companion animals. The remaining 56 veterinarians switched to different branches.

Of the 905 veterinarians, 366 (40%) had their own practice, 449 (49.6%) were employed, 25 (2.7%) were independent contractors and 27 (3%) were engaged in a combination of their own practice and employment; the remaining 38 were incapacitated, retired or did not answer this question. The veterinarians with their own practice were in most cases those in graduation groups 1 and 2; those employed were in graduation groups 2 and 3 (*χ* ^2^[df = 8, 278], *p* < 0.001).

### Prior knowledge of the disciplinary system

All 905 veterinarians had knowledge of the DS. Of these, 44 (5%) had knowledge prior to starting their veterinary training, 631 (70%) learned about it during their training and 230 (25%) after graduation. Almost all veterinarians who graduated in the last period had knowledge of DSs before graduating. By comparison, the oldest graduates acquired knowledge of the DS after graduation (*χ* ^2^[df = 2, 15], *p* < 0.001). On the question about whether the existence of a DS had influenced their way of practicing, 296 (32.8%) replied that it had not; 607 out of 903 (67.2%) replied that it had influenced their way of working; 370 (41%) replied that they had learned from published cases and had changed their way of working accordingly; 311 (34%) replied that they had become more informative towards their clients; and 191 (21%) indicated that they had started working in a more protocol‐based manner. For 121 (13%) veterinarians, additional remarks were provided that varied from using more defensive work practices, performing more diagnostic tests, avoiding difficult owners and making extensive notes in the medical records to avoid eventual complaints.

Of those who answered that it had not influenced their way of working, 40% were male and 60% were female. The male veterinarians were more inclined to say it had not influenced their way of working compared to the females (*χ* ^2^[df = 2, 7], *p* = 0.03). However, as reported earlier, most males graduated in earlier periods.

When asked whether they had actively obtained knowledge about DCs, 129 (14%) responded they had not, while 776 (86%) responded they had. Of those who had not educated themselves about DCs, 69 out of 905 (8%) indicated that it had not changed their way of working. In the group that had educated themselves about DCs, 549 out of 776 (61%) responded that they had adapted their way of working (*χ* ^2^[df = 6, 42], *p* < 0.001). The source used for gathering knowledge was mainly the Dutch veterinary journal (512 people), the internet (123 people) or a combination of these two (109 people).

### Disciplinary cases

The number of veterinarians who indicated having had to deal with one or more DCs was 381 (42%). Of this group, 249 had one case, 103 had two cases, 17 had three cases and 12 had more than four DCs (Table 1). Veterinarians in the older age groups were more often faced with more than one DC (Table 2).

**TABLE 1:** Distribution of disciplinary cases experienced according to the graduation period.

Disciplinary case	Graduation period 1 (before 1990)	Graduation period 2 (1991–2005)	Graduation period 3 (2006 onwards)	Total
Yes	121 (64%)	168 (49%)	87 (23%)	376
No	66 (35%)	174 (51%)	280 (67%)	520
Total	187	342	367	896

*Note*: Most disciplinary cases were experienced in the first two graduation periods (1 and 2) compared with period 3 (*χ* ^2^[df = 2, 97], *p* < 0.001).

**TABLE 2:** Distribution of disciplinary cases experienced according to age group.

Disciplinary case	Age group 1 (<30 years)	Age group 2 (30–39 years)	Age group 3 (40–49 years)	Age group 4 (50–59 years)	Age group 5 (>60 years)	Total
Yes	6	81	116	111	62	376
No	82	198	132	70	38	520
Total	88	279	248	181	100	896

*Note*: The number of veterinarians was divided into five age groups and whether or not they experienced a disciplinary case, the younger age groups were significantly different compared to the older age groups (*χ* ^2^[df = 4, 110], *p* < 0.001).

Veterinarians who had acquired knowledge prior to or during their training had faced fewer DCs than those who acquired it after graduation (Table 3). A similar effect was noted if the respondents had actively sought out knowledge about DCs or not. Veterinarians who had actively gained knowledge of DCs were less likely to be faced with a DC (*χ* ^2^[df = 1, 6], *p* = 0.015).

**TABLE 3:** Distribution of disciplinary cases according to when veterinarians acquired knowledge about the disciplinary system.

Disciplinary case	Prior to training/study	During training/study	After graduation	Total
Yes	15	226	140	381
No	29	405	90	524
Total	44	631	230	905

*Note*: Veterinarians who had acquired knowledge about the disciplinary system prior to and during their training appeared to have less disciplinary cases compared to those that acquired this knowledge after graduation (*χ* ^2^[df = 2, 45], *p* < 0.001).

A small number of veterinarians (*n* = 132) had to face two or more DCs. Facing a DC did not reduce the likelihood of getting a second or third DC (*χ* ^2^[df = 3, 3.7], *p* = 0.294).

Most of the respondents indicated that they worked as companion animal practitioners. There was no correlation between what field of veterinary medicine they practiced and whether or not they had to face a DC. The DCs were equally divided between the different disciplines within veterinary medicine (*χ* ^2^[df = 4, 3.8], *p* = 0.431). There was a difference in who filed the DC. For companion animals, it was predominantly the owner, while in mixed or large livestock work, the CS tended to file the DC (*χ* ^2^[df = 8, 94], *p* < 0.001).

For the veterinarians who had a DC (381, 42%), after the official procedure and hearing, one was inadmissible and 244 (64%) were unfounded. Hence, 136 rulings (36%) were judged to have merit. Of these 136 legitimate cases, 28 were founded and no penalty was applied, 66 received a warning or reprimand, 10 received a fine and one was given a fine with an unconditional suspension. This question was not answered by 31 respondents. Of 97 DCs, the respondents let us know the reason for the verdict. In 28 cases, the VDC concluded that inappropriate health care was given, in 31 cases, the wrong diagnosis was made, in eight cases, the provided care was not decisive enough and in 19 cases, the information about the disease or after care was insufficient. In 11 DCs, the communication with the client was not adequate. The number of veterinarians who disagreed with the ruling was 57 of 97 (59%), 19 were neutral and 20 agreed with the ruling.

Some DCs were also submitted to VAC (69/381, 18%). In 29 DCs, the verdict remained the same, in eight DCs, it became more severe and in 22 DCs, it was less severe compared to the VDC verdict.

Those veterinarians faced with a VDC were asked whether it had changed their way of working. In total, 103 (36%) said no, 40 (14%) were neutral and 143 (50%) said it had changed their way of working. Comparable numbers were seen for the DC that were judged founded: 31 (33%) said no, 16 (17%) were neutral and 46 (49%) changed their way of working.

The procedure length of these DCs varied, with 18% taking less than 6 months, 42% taking 6 months–1 year, 29% taking 1–1.5 years and 11% taking over 1.5 years.

There was no correlation between age group and ruling (Table 4). There appeared to be a correlation between the ruling and employment status (Table 5). This effect was also present for the complete group of 905 veterinarians, with 51% (187 out of 366) of the private practice owners having to face a DC and 49% did not. Of the veterinarians who were employed, only 33% faced a DC (149 out of 449) and 67% did not (*χ* ^2^[df = 3, 16.9], *p* < 0.001).

**TABLE 4:** Disciplinary case ruling by age group.

Ruling	Age group 1 (<30 years)	Age group 2 (30–39 years)	Age group 3 (40–49 years)	Age group 4 (50–59 years)	Age group 5 (>60 years)	Total
Unfounded	35	83	58	23	12	211
Founded	16	31	21	8	11	87
Total	51	114	79	31	23	298

*Note*: There was no correlation between ruling and age group (*χ* ^2^[df = 3, 4.6], *p* = 0.327).

**TABLE 5:** Disciplinary case ruling by employment status.

Ruling	Private practice owner	Employed	Combination	Independent contractor	Total
Unfounded	107 (58%)	114 (78%)	10 (83%)	10 (83%)	241
Founded	77 (42%)	33 (22%)	2 (17%)	2 (17%)	114
Total	184	147	12	12	355

*Note*: Veterinarians with their own private practice had a higher number of disciplinary cases, they also had a higher number of founded disciplinary cases, compared to veterinarians who were employed or who were independent contractors (*χ* ^2^[df = 3, 17.2], *p* < 0.002).

### Outcome and recommendations

For 306 of 381 veterinarians who had to face a DC shared how they experienced the complete procedure. Three veterinarians indicated that they terminated their job because they were unable to cope with the work anymore, 41 (11%) felt sick and had considered stopping work, 219 (57%) found it very time consuming and stressful, 23 (6%) said it had influenced them slightly but not severely and 20 (5%) said it had not bothered them at all. For 46 of 357 (13%), they indicated that they had not received any support from colleagues, friends or family. While 311 (87%) indicated that they did receive support, in most cases, it was from their family, friends or colleagues. Only five veterinarians indicated that they had asked for legal advice.

All veterinarians were asked to provide recommendations to improve the DS. Of the complete group of 905 veterinarians, 402 had no recommendations. The remaining veterinarians suggested a court fee for the complaining party (*n* = 318 out of 503, 63%), mediation (*n* = 198, 39%), shorter procedure (*n* = 273, 54%), use of a zoom (Zoom.us) or teams (Microsoft Teams) (*n* = 287, 57%) and pre‐screening by an experienced chairperson (*n* = 229, 45%).

### Statement questions

Four statement questions were formulated. A total of 67% of the veterinarians agreed that the government wanted to promote good veterinary practice (GVP) with the installation of a DS, while 56% of veterinarians agreed that this indeed succeeded (statement question 2). This difference was statistically significant (*χ* ^2^[df = 16, 650], *p* < 0.001). Approximately 70% of veterinarians were neutral or felt that they could learn from the DS, while 30% felt that it only served the complainer (Table 6).

**TABLE 6:** Responses to statement questions about good veterinary practice (GVP) and the disciplinary system (DS).

Statement	Totally disagree	Disagree	Neutral	Agree	Totally agree	Number
1. The government wanted to promote GVP, do you agree?	35	84	152	440	115	826
2. The DS has as a main goal GVP, is this the case?	64	174	128	401	64	831
3. We can learn from the DS	51	131	143	426	73	824
4. The DS only serves the complainer	69	178	202	315	67	831

### The recommendations of the participants

Veterinarians could choose to discontinue the DS, to do nothing or to vote for revision. Only 13% wanted it to be discontinued, 10% said that it was functioning perfectly and 77% recommended revisions. There was no correlation between having faced a DC and this answer (*χ* ^2^[df = 2, 5.85], *p* = 0.054).

## DISCUSSION

To the best of the authors’ knowledge, this is the first detailed study of the DS in a large group of Dutch veterinarians. More than 20% of the total number of Dutch working veterinarians filled in the questionnaire. Our results suggest that older (male) graduates faced more DCs. The same applies for veterinarians with their own private practice: they had more DCs than those who were employed. However, the majority of veterinarians with a private practice are older (male) graduates. In 1980, 95% of the graduates were male. From 1981 to 1990, the graduates were 50% male and 50% female; the most recent survey reported that 80% of graduates were female.^18^ A more logical and possible reason for this effect is just the number of years the respondent has worked as a veterinarian. A similar observation was reported in a study that looked at the effect of CCs on small animal internists.^4^ In this study, the associates with 10–19 years of practice had more CCs than the other groups.

Interestingly, veterinarians who actively learned about DCs (this group was not influenced by gender, age or graduation period) faced fewer DCs than those who did not. Furthermore, the veterinarians who adapted their way of working had fewer DCs than those who did not. There was some bias in this last observation because most older veterinarians had indicated that they had not changed their way of working after the introduction of the DS. This finding is offset by the fact that if a veterinarian educated themself (and applied this knowledge), it reduced the risk of facing a DC. The majority of veterinarians changed their way of working after having faced a DC. The only group that did not were the veterinarians who faced multiple DCs; in this group, there was apparently no learning effect. In one study, 71.7% of small animal internists changed their way of working, which aligns with the 67.2% observed in our study.^4^

Veterinarians working in the field of companion animal medicine had the same risk of receiving a DC as veterinarians working in other fields, such as large animal livestock. The only difference was who filed the complaint. Companion animal veterinarians had CCs and large animal veterinarians received complaints from CSs. Communication was mentioned as a reason in 21% and veterinary care/mistakes was given as a reason in 69% of the DCs. Learning from earlier cases, explaining everything in more detail and utilising a more protocol‐based practice will help prevent DCs. In this group of veterinarians, 67% adopted these methods. Thirteen percent responded that they avoided difficult owners, performed more diagnostics and made more extensive notes in the medical records. This is an example of defensive medicine and it will, as in human medicine, lead to higher costs.^11^, ^13^, ^19^

We could not determine whether there was an effect of graduating from Ghent University or Utrecht University. At the time the survey was first sent out (2017), it was unknown how many veterinarians came from Ghent University. It was wrongfully estimated to be low and for this reason, it was not included in the questionnaire. Subsequently, it was reported that approximately 80% were Utrecht graduates and 20% were Ghent graduates.^16^ Furthermore, more exact figures were published after completion of our study.^18^

In our study, 12% of the veterinarians who had faced a DC wanted to stop practicing, stopped practicing or felt sick. Furthermore, 57% found it time consuming and stressful. These figures are in line with previous studies.^4^, ^5^ Although the respondents indicated that a DC has a severe impact on their professional wellbeing, only five veterinarians indicated that they had sought legal advice.^12^ This is in contrast with Dutch medical doctors, where 93% indicated that they had obtained legal support when faced with a DC.^12^ While getting support from colleagues and family is very important and helps one cope with a DC,^20^ it may also be prudent for veterinarians to seek legal support.

The answers to the statement questions demonstrate that the majority of veterinarians agreed that a DS could promote GVP. They agreed that veterinarians could learn from it, although a large proportion stated that it primarily served the person(s) who filed the complaint. Given that 77% of the veterinarians proposed adjustments, it seems safe to conclude that the current DS is not functioning optimally. One could even say that CCs apparently do not contribute to a safe working environment for most private practitioners. Similar statements have been made after analyses of human medical DCs.^12^, ^20^, ^21^, ^22^, ^23^ It has been recommended that it would be better to begin the process with a round table talk before continuing with the veterinary disciplinary hearing.^21^

The participant recommendations were in line with different, maybe better functioning DSs, such as those in the UK and USA.^3^, ^6^ Even in these countries, the impact of DCs on veterinarians can be significant.^4^, ^5^, ^24^ Given that 244 of 381 DCs were judged to be unfounded, one could ask how many could have been avoided if an experienced chairperson had pre‐screened them, if mediation had been an option or if a court fee was required.

Although the number of respondents was high, there were clear limitations. The first is that we send out a call for this survey twice with a period in between of nearly 5 years. When we had sent out the first call, we felt the number of participants was not enough; consequently, it was sent out a second time. Veterinarians were informed about this; even so, we cannot fully exclude that some participated twice. Second, the number of practitioners was 870 out of 905 (96%). The questionnaire was sent out to all veterinarians in the Netherlands. According to a previous study, it was estimated that 75% of all veterinarians were practitioners.^18^ Hence, the number of practitioners is overrepresented. Third, an unknown number of participants studied in Belgium. This study did not provide the information to account for this. We used three time periods that addressed the Dutch situation. Belgium graduates had been educated about best practices and the DS since 1950, so this division in the three time periods should not be applied to those graduates.

Based on a previous study,^18^ the number of veterinarians working within the field of companion animal medicine is not overrepresented. In our study, the number of companion animal practitioners was 78%, similar to a previous study.^18^ It is possible that veterinarians who faced a DC were more inclined to participate. In our study, 42% of the participants had faced a DC. If this number is representative for practitioners, the total number of DCs would be 1800 for the complete time period of nearly 60 years. In a previous study, we analysed nearly 1200 DCs within a time period of 15 years.^1^ This may indicate that the group of participants is, in this instance, representative.

The questionnaire was not validated for mistakes in answering. However, we feel that the result provides sufficient material to critically reflect on the Dutch DS.

This study demonstrated that CCs had an impact on veterinarians. In total, 69% of the veterinarians who faced a DC indicated that they had stopped, had considered stopping, had felt sick or had experienced it as time consuming and stressful, despite the fact that only 36% of the DCs filed were judged to be founded. Recommendations to reduce stress and improve the procedure included: (1) shortening the length of the procedure, (2) screening for validity, (3) using zoom/teams for communication with the VDC, (4) the option of mediation before getting involved in a full procedure and (5) instituting a complaint fee.

## AUTHOR CONTRIBUTIONS

Iaira E. Boissevain and Paul J.J. Mandigers were responsible for conception of the study, acquisition of the data, analysis and interpretation, the statistical analysis and writing—review and editing of the manuscript. Anthonie W. Jongbloed, Franck L.B. Meijboom and Jan Willem Hesselink were responsible for writing—review and editing of the manuscript. All authors contributed to the article and approved the submitted version.

## CONFLICTS OF INTEREST STATEMENT

The authors declare they have no conflicts of interest.

## FUNDING INFORMATION

The authors received no specific funding for this work.

## ETHICS STATEMENT

The authors confirm that the ethical policies of the journal, as noted on the journal's author guidelines page, have been adhered to. No ethical approval was formally required, given that all veterinarians who participated were asked, before starting with the survey, to agree to participate and allow use of the submitted information for research. All responses were processed anonymously, as the IP address or any information that could identify them as a person, was not stored.

## Supporting information

Supporting information
Questionnaire S1

## Data Availability

Raw data are available on reasonable request.

## References

[1] Boissevain IE , van Rooij M , Jongbloed AW , Meijboom FLB , Hesselink JW , Mandigers PJJ . 15 years of facts and figures on veterinary disciplinary measures in the Netherlands. Front Vet Sci. 2022;9:987797. 10.3389/fvets.2022.987797 36439334 PMC9691658

[2] Boissevain I , Boerema L . Recht uit de dierenartsenpraktijk—dierenartsen, clienten, patienten en het recht (Straight from the veterinary practice—veterinarians, clients, patients and the law). Den Haag, The Netherlands: SDU Publishers; 2004. p. 5–142.

[3] RCVS. Veterinary Disciplinary System RVCS. 2022. Available from: https://www.rcvs.org.uk/concerns/disciplinary‐hearings/. Accessed 7 June 2023

[4] Bryce AR , Rossi TA , Tansey C , Murphy RA , Murphy LA , Nakamura RK . Effect of client complaints on small animal veterinary internists. J Small Anim Pract. 2019;60:167–72. 10.1111/jsap.12936 30284723

[5] Rogers CW , Murphy LA , Murphy RA , Malouf KA , Natsume RE , Ward BD , et al. An analysis of client complaints and their effects on veterinary support staff. Vet Med Sci. 2022;8:925–34. 10.1002/vms3.725 35044103 PMC8959328

[6] Labriola J , Garabed R , Sinclair C , Marsh AE . Insights from veterinary disciplinary actions in California 2017–2019. Front Vet Sci. 2021;8:786265. 10.3389/fvets.2021.786265 35004928 PMC8734430

[7] Balch CM , Oreskovich MR , Dyrbye LN , Colaiano JM , Satele DV , Sloan JA , et al. Personal consequences of malpractice lawsuits on American surgeons. J Am Coll Surg. 2011;213:657–67. 10.1016/j.jamcollsurg.2011.08.005 21890381

[8] Hayes GM , LaLonde‐Paul DF , Perret JL , Steele A , McConkey M , Lane WG , et al. Investigation of burnout syndrome and job‐related risk factors in veterinary technicians in specialty teaching hospitals: a multicenter cross‐sectional study. J Vet Emerg Crit Care. 2020;30:18–27. 10.1111/vec.12916 PMC7003767 31840933

[9] Bourne T , Wynants L , Peters M , Van Audenhove C , Timmerman D , Van Calster B , et al. The impact of complaints procedures on the welfare, health and clinical practise of 7926 doctors in the UK: a cross‐sectional survey. BMJ Open. 2015;5:e006687. 10.1136/bmjopen-2014-006687 PMC4316558 25592686

[10] Wu AW . Medical error: the second victim. The doctor who makes the mistake needs help too. BMJ. 2000;320:726–7. 10.1136/bmj.320.7237.726 10720336 PMC1117748

[11] Studdert DM , Mello MM , Sage WM , DesRoches CM , Peugh J , Zapert K , et al. Defensive medicine among high‐risk specialist physicians in a volatile malpractice environment. JAMA. 2005;293:2609–17. 10.1001/jama.293.21.2609 15928282

[12] Laarman BS , Bouwman RJ , de Veer AJ , Hendriks M , Friele RD . How do doctors in the Netherlands perceive the impact of disciplinary procedures and disclosure of disciplinary measures on their professional practice, health and career opportunities? A questionnaire among medical doctors who received a disciplinary measure. BMJ Open. 2019;9:e023576. 10.1136/bmjopen-2018-023576 PMC6429727 30878977

[13] Anderson RE . Billions for defense: the pervasive nature of defensive medicine. Arch Intern Med. 1999;159:2399–402. 10.1001/archinte.159.20.2399 10665887

[14] Huo L , Zhou Y , Li S , Ning Y , Zeng L , Liu Z , et al. Burnout and its relationship with depressive symptoms in medical staff during the COVID‐19 epidemic in China. Front Psychol. 2021;12:616369. 10.3389/fpsyg.2021.616369 33746836 PMC7969997

[15] Sonneveld D , Goverts Y , Duijn C , Camps G , Bougie R , Mastenbroek N . Dutch veterinary graduates leaving practice: a mixed‐methods analysis of frequency and underlying reasons. Vet Rec. 2023;192:e2178. 10.1002/vetr.2178 36056552

[16] Bergevoet RHM , Benus M , van der Valk O . Een tekort aan dierenartsen in Nederland? Een eerste inventarisatie (A shortage of veterinarians in the Netherlands? A first inventory). Contract No. 2020‐119. Wageningen University & Research; 2020. 10.18174/534170

[17] Verhoef LM , Weenink JW , Winters S , Robben PB , Westert GP , Kool RB . The disciplined healthcare professional: a qualitative interview study on the impact of the disciplinary process and imposed measures in the Netherlands. BMJ Open. 2015;5:e009275. 10.1136/bmjopen-2015-009275 PMC4663436 26608639

[18] van Vuuren D , Vlaanderen M , Pomp M , Geelen J . De arbeidsmarkt voor dierenartsen. Knelpunten en Perspectieven (The labour market for veterinarians. Bottlenecks and perspectives). Contract No. 2022‐69. Amsterdam, Netherlands: Geelen Consultancy; 2023. Available from: https://www.seo.nl/publicaties/de‐arbeidsmarkt‐voor‐dierenartsen/. Accessed 7 June 2023.

[19] Summerton N . Positive and negative factors in defensive medicine: a questionnaire study of general practitioners. BMJ. 1995;310:27–9. 10.1136/bmj.310.6971.27 7827550 PMC2548439

[20] Laarman BS , Bouwman RJR , de Veer AJE , Friele RD . Is the perceived impact of disciplinary procedures on medical doctors' professional practice associated with working in an open culture and feeling supported? A questionnaire among medical doctors in the Netherlands who have been disciplined. BMJ Open. 2020;10:e036922. 10.1136/bmjopen-2020-036922 PMC7692813 33243787

[21] Legemaate J . Omdenken over het tuchtrecht (Rethinking about disciplinary law). Tijdschrift voor Gezondheidsrecht. 2021;45:2. 10.5553/TvGR/016508742021045001001

[22] Giard RW . [In Dutch—The Medical Code of Practice: clear reasons for existing, benefit less certain]. Ned Tijdschr Geneeskd. 2006;150:2830–2. 17216733

[23] Hendriks AC . [In Dutch—The WKKGZ, a new act with far‐reaching consequences for physicians]. Ned Tijdschr Geneeskd. 2015;159:A9799. 26732222

[24] Babcock S , Mantese T , Pfeiffer CL . Effects of veterinary board disciplinary actions on veterinarians licensed in multiple states. J Am Vet Med Assoc. 2005;227:1906–9. 10.2460/javma.2005.227.1906 16379625

