# Supplementary material for: Experiences and recommendations of veterinarians for the Dutch disciplinary system—a survey‐based study
Source: Vet Rec Open. 2023 Jun 25;10(1):e67. doi: 10.1002/vro2.67 (PMC10290824; doi:10.1002/vro2.67)
Supplement: Supplementary file 1 — Supporting information Questionnaire S1 [file VRO2-10-e67-s001.pdf]

Supporting information Questionnaire S1

Dear colleague,

Thank you for your participation. By clicking on the 'proceed' button you agree to participate and allow us to use your answers for this study investigating the Dutch veterinary disciplinary system and what its effect is on you as a professional.

This study is part of the PhD study of Mrs. Iaira Boissevain (LLM, BSc lawyer and specializing in veterinary disciplinary system). Her mentor is Dr. Paul Mandigers.

This questionnaire is quite simple. It is divided into four sections of which the third is only applicable for you if you ever had to face a disciplinary case. If not, you will automatically move to the last part of this questionnaire where we have placed a few statement questions. Please answer all questions. If a question is not applicable for you, you can click on the button 'next' and you will move to the following question. However, a small number of questions are obligatory. If you do not answer the obligatory question you cannot proceed. To complete this survey, you need 9 to 20 minutes.

We kindly ask you to fill in this questionnaire only once. Your answers will be registered with a unique code. However, we do not store your IP address or any other information that can be used to Identify you as a person. Your responses will be anonymous and untraceable. In the event you disclose any personal information we will not disclose this.

[remark: Obligatory questions are marked with an asterisk]

*Proceed button --> by clicking you agree to participate and allow us to use your answers.

[Demographic part]

Q1 What is your gender?
- Male / - Female / - non-binary / - prefer not to say

Q2 What is your graduation year?

*Q3 Are you, at this moment, working within the field of veterinary medicine?
1 Yes / 2 No

Q4 If you are no longer active within the field of veterinary medicine: what is the reason?
1 retired / 2 incapacitated / 3 other work field / 4 other, namely

*Q5 In what kind of sector are you working as a veterinarian? Multiple answers are possible.
1 Companion animals / 2 Large animals / 3 Horses / 4 Mixed practice / 5 zoo / 6 pharmacy / 7 food industry / 8 government / 9 education / 10 management / 11 others

Q6 Have you worked prior to you current job in another field?
1 Yes / 2 No

*Q7 What is your current employment situation?
1 Own practice / 2 salaried employment / 3 combination of 1 and 2 / 4 independent contractor / 5 other namely

[Awareness of the existence of the VDC and VAC - influence on practicing]

*Q8 In the Netherlands our government has instated a veterinary disciplinary council (VDC) and veterinary appeal council (VAC). Are you aware of this situation?
1 Yes / 2 No

*Q9 When did you learn of this veterinary disciplinary council?
1 Prior to my study / 2 During my veterinary study / 3 after my graduation

*Q10 Has the establishment of this VDC and VAC changed the way you practice? This question is not about any case that was filed against you. Multiple answers are possible.
1 No / 2 Yes I am using a more protocol based way of working / 3 I discuss everything with the owner / 4 I have learned from a verdict of the council and avoid mistakes made by others / 5 other namely

Q11 How do you acquire knowledge of rulings of the council? Multiple answers are possible.
1 I do not / 2 Internet / 3 Veterinary journal / 4 other namely

Q12 If you are reading the verdicts of the council. Has this influenced the way you practice? Multiple answers are possible.
1 No / 2 Yes I am using a more protocol based way of working / 3 I discuss everything with the owner / 4 I have learned from a verdict of the council and avoid mistakes made by others / 5 other namely

[Section addressing veterinarians that had to experience a disciplinary case]

*Q13 Have you ever had a VDC case filed against you?
1 Yes / 2 No

*Q14 Has the establishment of this VDC, even if you have never had a case filed against you, changed the way you practice?
1 Yes / 2 No *[**CONDITION**: If Q13 was **Yes** the survey will run from Q13 to Q30] & [If Q13 was **No** the survey will move after Q14 to Q31]*

*Q15 If you have had a case filed against you: how often has this been?
1 Once / 2 Twice / 3 Thrice / 4 four or more cases

For answering the following questions, please use your first disciplinary case

Q16 What was your age when this case was filed?

Q17 At that time were you working in the same field as answered earlier or was it something else?
1 Yes / 2 No I was working elsewhere namely,

Q18 Who filed the disciplinary case against you?
1 the owner / 2 civil servant / 3 other namely

Q19 How long has this patient been under your care?
1 it was an emergency case / 2 I saw it for the first time; it wasn't an emergency case / 3 I knew the animal less than 4 weeks / I knew the animal less than 1 to 3 months / I knew the animal already for 3 to 6 months / I knew the animal already for over 6 months / I knew the animal already over a year

Q20 What was the verdict of the VDC?
1 Unfounded / 2 Founded but no disciplinary measure / 3 warning / 4 reprimand / 5 fine / 6 fine and conditional expulsion / 7 partial or complete expulsion to practice for a maximum of one year / 8 partial or complete expulsion to practice

Q21 Has the VDC provided a reason?
1 insufficient health case / 2 wrong diagnosis and-or treatment / 3 arrived too late or not at all / 4 nor referral or referral on time / 5 incomplete information about the diagnosis and-or treatment etc. / 6 other namely

Q22 Do you think that the VDC ruled correctly in your case?

1 Total disagree / 2 disagree / 3 neutral / 4 agree / 5 total agree

Q23 Can you elaborate?

Q24 Has you case also been submitted to the VAC?
1 Yes / 2 No

Q25 Did the VAC change the verdict?
1 No / 2 less severe / 3 more severe

Q26 Do you think that the VAC ruled correctly in your case?
1 Yes / 2 No, namely

Q27 Did you, after this case that was filed against you changed the way you practice?
1 Total disagree / 2 disagree / 3 neutral / 4 agree / 5 totally agree

Q28 How long did the complete procedure take place?
1 Less than 6 months / 2 6 months to a year / 3 one year to one-and-a-half years / 4 one-and-a-half years to two years / 5 over 5 years

Q29 How did you experience this disciplinary case?
1 I stopped working / 2 I really felt bad and considered stopping / 3 frustration. I took a lot of energy and time / 4 it was important, but I didn't stay awake over it / 5 it didn't bother me at all

Q30 Did you get any (emotional) support? Multiple answers are possible
1. No / 2 yes from my family / 3 yes from my colleagues / 4 yes from friends / 5 other namely

[Statement questions]

Q31 Can you recommend anything to changes this system:
1 court fee / 2 mediation / 3 shorten the procedure and use written rounds / 4 let a chairman / expert deal with minor cases / 5 other namely

Q32 The goal of our government was to construct a disciplinary system to promote good veterinary practice.
1 Total disagree / 2 disagree / 3 neutral / 4 agree / 5 total agree

Q33 The goal of a disciplinary system is to promote good veterinary practice. Is this your opinion?
1 Total disagree / 2 disagree / 3 neutral / 4 agree / 5 total agree

Q34 The goal of the disciplinary system is that we can learn from it and improve our way of working
1 Total disagree / 2 disagree / 3 neutral / 4 agree / 5 total agree

Q35 The goal of the disciplinary system is just to satisfy disgruntled owners
1 Total disagree / 2 disagree / 3 neutral / 4 agree / 5 total agree

Q36
What did you change in your daily work after dealing with disciplinary case? Open question

Q37 If you were allowed to choose. Shall we abolish the disciplinary system?
1 Yes stop it / 2 No it works fine / 3 No but please change it

Q38 Any remarks? Open question
